# Mechanism of Cullin3 E3 Ubiquitin Ligase Dimerization

**DOI:** 10.1371/journal.pone.0041350

**Published:** 2012-07-20

**Authors:** Yin Yin Choo, Thilo Hagen

**Affiliations:** Department of Biochemistry, Yong Loo Lin School of Medicine, National University of Singapore, Singapore, Singapore; University of Oldenburg, Germany

## Abstract

Cullin E3 ligases are the largest family of ubiquitin ligases with diverse cellular functions. One of seven cullin proteins serves as a scaffold protein for the assembly of the multisubunit ubiquitin ligase complex. Cullin binds the RING domain protein Rbx1/Rbx2 via its C-terminus and a cullin-specific substrate adaptor protein via its N-terminus. In the Cul3 ubiquitin ligase complex, Cul3 substrate receptors contain a BTB/POZ domain. Several studies have established that Cul3-based E3 ubiquitin ligases exist in a dimeric state which is required for binding of a number of substrates and has been suggested to promote ubiquitin transfer. In two different models, Cul3 has been proposed to dimerize either via BTB/POZ domain dependent substrate receptor homodimerization or via direct interaction between two Cul3 proteins that is mediated by Nedd8 modification of one of the dimerization partners. In this study, we show that the majority of the Cul3 proteins in cells exist as dimers or multimers and that Cul3 self-association is mediated via the Cul3 N-terminus while the Cul3 C-terminus is not required. Furthermore, we show that Cul3 self-association is independent of its modification with Nedd8. Our results provide evidence for BTB substrate receptor dependent Cul3 dimerization which is likely to play an important role in promoting substrate ubiquitination.

## Introduction

Cullin3 (Cul3) E3 ubiquitin ligases are involved in the recognition and recruitment of numerous important substrates for ubiquitination [1]–[4]. Cul3 dependent ubiquitination has emerged as a mechanism to control various critical cellular processes including the antioxidant response, cell migration, cell cycle progression and retrograde trafficking [3], [5]–[8]. It has been shown that lack of Cul3 is embryonically lethal in mice [9]. Functionally, absence of Cul3 has been reported to cause inhibition of cell migration in human and drosophila cells [3]. This is due to stabilization of the Cul3 substrate RhoA which controls actin cytoskeleton stress fiber development [3]. As a consequence, cells with reduced Cul3 expression exhibit abnormal actin stress fibers and distorted cell morphology [3].

Cul3 is a member of the cullin protein family, which also includes Cul1, 2, 4A, 4B, 5 and 7 in mammalian cells [10]–[11]. Similar to other cullins, Cul3 serves as a scaffold protein for the assembly of the multisubunit ubiquitin ligase complex that contains a RING domain protein and a substrate adaptor protein [10], [12]. All Cul3 substrate receptors contain a “Bric a brac, Tram-track and Broad Complex/Pox virus and Zinc finger” (BTB/POZ) domain, which binds to the Cul3 N-terminus. Cul3 recruits substrates via a variable substrate binding domain such as Zinc Finger, Kelch, MATH and Ras homology domains [13]. Cul3 binds to the RING domain protein Rbx1 via its C- terminus. Rbx1 functions to recruit the ubiquitin-charged E2 conjugating enzyme [10]–[11]. The ubiquitination activity of all cullin E3 ligase complexes is activated by conjugation of the ubiquitin-like protein Nedd8 onto a conserved C-terminal lysine residue in the cullin protein [14]. This neddylation process is mediated via the Nedd8-specific E1 APPBPA-Uba1 heterodimeric enzyme and the E2 enzyme Ubc12. Nedd8 is known to activate all Cullin ubiquitin ligases. The Nedd8 conjugation activates Cullin RING E3 ligases by inducing a conformational change in the cullin C-terminus/Rbx1 structure, thereby increasing the flexibility of the Rbx1 RING domain. Thus, this brings the ubiquitin charged E2-conjugating enzyme and the target substrate into a close proximity [14]–[16].

The best-characterized BTB domain containing Cul3 substrate receptors are SPOP and Keap1. The substrate binding domain of SPOP comprises of a MATH domain whereas Keap1 contains a Kelch repeat domain [13], [17]–[18]. Both the MATH domain and the Kelch repeat domains play an essential role in recruiting Cul3 target substrates for degradation [17]–[18]. It is also known that Cul3 substrate receptor proteins form tightly bound homodimers via their BTB domains [13]. Keap1 protein homodimerizes to recognize two separate motifs, the ETGE and DLG motifs, in its substrate Nrf2 [19]–[20]. The binding of the Nrf2 transcription factor via two binding sites to the Kelch domains of the Keap1 homodimer is important to position Nrf2 in an appropriate orientation to facilitate ubiquitin transfer from the ubiquitin-charged E2 enzyme [19]–[21]. The ETGE motif serves as a “hinge” to clutch on to Keap1 and mediates the high affinity binding between Nrf2 and Keap1 whereas the DLG motif in Nrf2 acts as a “latch” to mediate the low affinity binding [20], [22]. This “hinge & latch” binding mode is essential for Nrf2 ubiquitination and Nrf2 ubiquitination is inhibited if binding of either the DLG or ETGE motif to Keap1 is disrupted [23]. Under normal conditions, the Cul3/Rbx1-Keap1 ligase recruits Nrf2 constitutively for polyubiquitination and consequently targets it for degradation to maintain low basal levels of the transcription factor [6], [24]. However, Nrf2 ubiquitination is inhibited upon exposure of cells to electrophiles and oxidative stress due to the suppression of the Cul3/Rbx1-Keap1 ligase activity. This leads to an increase in Nrf2 stability and hence the activation of the antioxidant transcriptional response [6], [24].

It has also been shown that a homodimeric SPOP substrate receptor complex facilitates the ubiquitination of Jun kinase phosphatase Puckered (PUC) and variant histone MacroH2A [17]. PUC and MacroH2A are substrates for the Cul3/Rbx1-SPOP E3 ligase in Drosophila and mammalian cells, respectively [4], [25]. These two substrates have multiple SPOP-binding sites to bind to the SPOP dimer [17]. A model has been proposed according to which ubiquitination of PUC or MacroH2A is mediated by binding of a single substrate to two flexibly orientated substrate-binding sites in the MATH domains of the SPOP dimer [17].

Dimerization of the Skp1-Cul1-F-box(SCF)^Cdc4^ complex via the cdc4 substrate receptor has been shown to promote the efficiency of substrate ubiquitination [26]. This is likely due to the dimeric Cullin ligase complex providing a bivalent geometry that has two docking sites for E2 ubiquitin enzyme and thus initiates optimal ubiquitin transfer and elongation of the target substrate [26]. With regards to the dimerization of the Cul3 E3 ligase, two models have been proposed. Firstly, it has been suggested that the Cul3 dimer complex formation is indirect and mediated via BTB domain-containing substrate receptor homodimerization [13]. Our previous study also provided support for the model that Cul3 dimer complex formation is mediated via the association with BTB domain-containing proteins [27]. In contrast, Wimuttisuk and Singer, (2007) reported that two Cul3 proteins dimerize directly via interaction of the WH-B domain. According to this model, the BTB domain-containing proteins are not required for Cul3 dimerization. The dimer consists of one neddylated Cul3 and one unneddylated Cul3 molecule [28]. In the current study, we studied the mechanism of Cul3 dimerization in mammalian cells in order to resolve this controversy. We also investigated the proportion of Cul3 that exists as a dimeric Cul3/Rbx1-BTB protein complex *in vivo*.

## Results

### Mutant Cullin3 proteins that are unable to bind to BTB domain-containing proteins exhibit markedly reduced Cul3-Cul3 association

**Figure 1:**
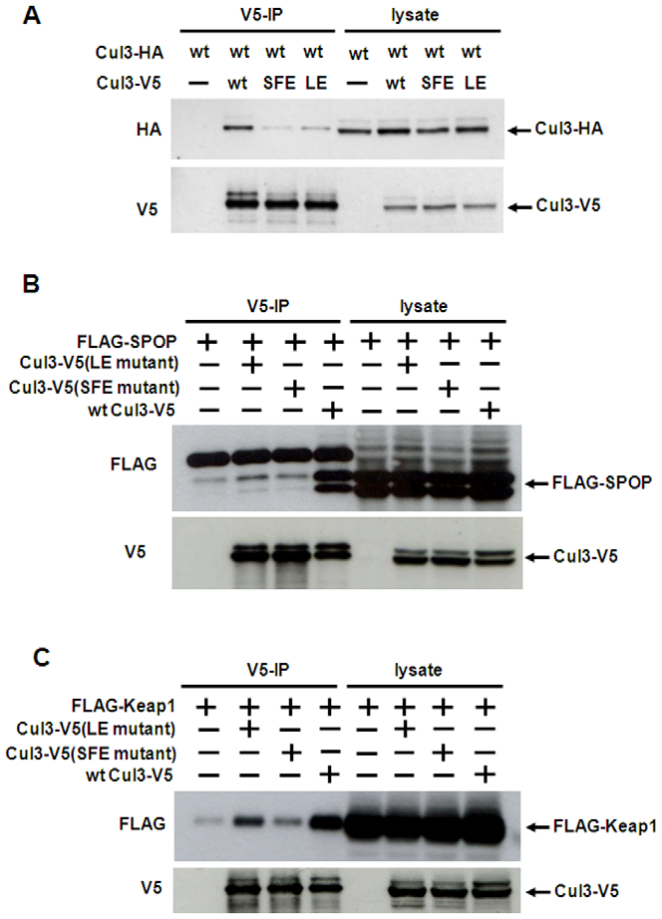
Mutant Cullin3 proteins that are unable to bind to substrate receptor subunits exhibit reduced Cul3 self-association. *A–C*, HEK293 cells were transfected in 60-mm cell culture plates for 2 days with expression constructs for the proteins indicated at the top of each panel. The cells were lysed, and the lysates were subjected to V5 immunoprecipitation (*IP*), as described under “Materials and Methods.” Immunoprecipitates and aliquots of the cell lysates were analyzed by Western blotting with the indicated antibodies.

To test whether Cul3-Cul3 binding is mediated via the homodimerization of BTB proteins, we previously used a Cul3 S53A/F54A/E55A mutant (Cul3(SFE)) that lacks the ability to bind to BTB proteins including Keap1 [27]. However, Wimuttisuk and Singer, (2007) used a different mutant of Cul3, L52AE55A (Cul3(LE)) [29], [30]. We therefore initially compared the ability of the two different Cul3 mutants to associate with wild type Cul3 by using co-immunoprecipitation assays. As shown in Figure 1*A*, both Cul3 mutants exhibited a marked decrease in the binding to wild type Cul3 although Cul3(LE) mutant showed a slightly higher remaining binding affinity to wild type Cul3. Our results that Cul3 mutants that are unable to bind to BTB domain-containing proteins show markedly reduced Cul3-Cul3 association suggest that homodimerization of BTB proteins is important for Cul3-Cul3 binding. These Cul3-Cul3 associations most likely represent Cul3 dimerization mediated by BTB dimers.

We next investigated the binding of these two Cul3 mutants to the BTB domain-containing proteins SPOP and Keap1. As shown in Figure 1*B*, both the Cul3(LE) and Cul3(SFE) mutants showed no significant interaction with the SPOP protein compared to strong binding between wild type Cul3 and SPOP. When measuring binding of Cul3 to Keap1, we also noted markedly reduced binding when using Cul3(SFE). In contrast, the interaction of Cul3(LE) with Keap1 was only moderately reduced compared to wild type Cul3, (Figure 1 *C* and *D*). Therefore, the Cul3(LE) and Cul3(SFE) mutants have different affinities to Keap1 and possibly to other BTB proteins. This may explain why association between Cul3 and Cul3(LE) was still observed in the study by Wimuttisuk and Singer, (2007).

### Cul3-Cul3 binding is independent of the WH-B domain

**Figure 2:**
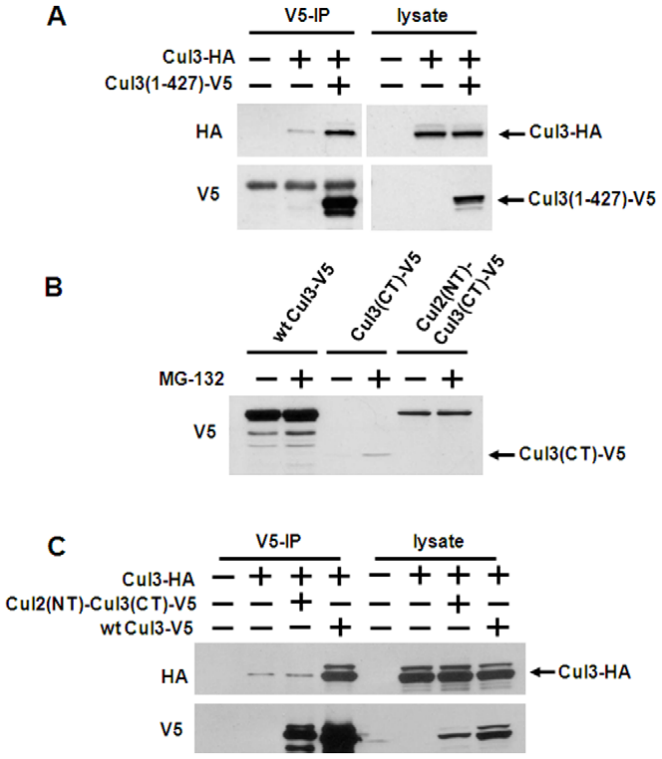
Cul3-Cul3 binding requires the Cul3 N-terminus but not the Cul3 C-terminus. *A*, The cells were cotransfected with Cul3-HA and Cul3(1–427)-V5 (Δ*CT*), as indicated at the top of each panel. The cells were lysed, and the lysates were subjected to V5 immunoprecipitation (*IP*), Immunoprecipitates and aliquots of the cell lysates were analyzed by Western blotting with the indicated antibodies. *B*, cells were transfected with wild type (*wt*) Cul3-V5, Cul3(CT)-V5 and Cul2(NT)-Cul3(CT)-V5, as indicated. Two days after transfection, the cells were treated with 25 ìM MG-132 for 6 h, where indicated, and then lysed, followed by Western blotting of cell lysates with V5 antibody. *C*, HEK293 cells were transfected in 60-mm cell culture plates for 2 days with expression constructs for the proteins as indicated at the top of each panel. After cell lysis, the cell lysates were subjected to V5-immunoprecipitation and immunoblotting with V5 and HA antibodies.

Wimuttisuk and Singer, 2007 proposed that in the active Cul3 ubiquitin ligase complex, the dimerization of Cul3 proteins is mediated via the hydrophobic residues in a Winged-Helix B (WH-B) domain near the Cul3 C-terminus. Thus, according to this model, the Cul3 C-terminus but not the Cul3 N-terminus is involved in the dimerization. To test for the involvement of the WH-B domain, we generated a C-terminal deletion construct of Cul3, comprising of amino acids 1–427 which excludes the WH-B domain in human Cul3, and measured its interaction with wild type Cul3 by co-immunoprecipitation assays. As shown in Figure 2*A*, Cul3(1–427) could still bind to wild type Cul3. To further confirm that Cul3-Cul3 association is not mediated via the Cul3 C-terminal WH-B domain, we generated an N-terminal deletion of Cul3, comprising of amino acids 392–768 containing the WH-B domain. However, this truncated Cul3 protein was not well expressed. This is partially due to N-terminally deleted Cul3 being degraded by the 26S proteasome (Figure 2*B*). Therefore, we generated a fusion construct comprised of the C-terminus of Cul3 (amino acid 392–768) fused to the N-terminus of Cul2 (amino acid 1–394) and also carrying a C-terminal V5-tag for immunoprecipitation (Cul2(NT)-Cul3(CT)-V5). As shown in Figure 2*B* lane 5 and lane 6, Cul2(NT)-Cul3(CT)-V5 was expressed well compared to Cul3(CT)-V5 and not subject to proteasome dependent degradation. We then used the Cul2(NT)-Cul3(CT)-V5 fusion construct to determine whether the Cul3 C-terminus could mediate Cul3-Cul3 association. As shown in Figure 2*C*, wild type Cul3-V5 could interact with full length Cul3-HA. In contrast, no interaction between the Cul2(NT)-Cul3(CT)-V5 fusion protein and Cul3-HA could be observed. These results suggest that the Cul3 N-terminus is necessary for Cul3-Cul3 binding, which is likely due to the requirements of the BTB domain-containing protein (eg, Keap1 or SPOP) for Cul3 self-association. In contrast, the WH-B domain near the Cul3 C-terminus does not play a role in Cul3-Cul3 association.

### Inhibition of Cullin Neddylation Does Not Affect Cul3-Cul3 Binding

**Figure 3:**
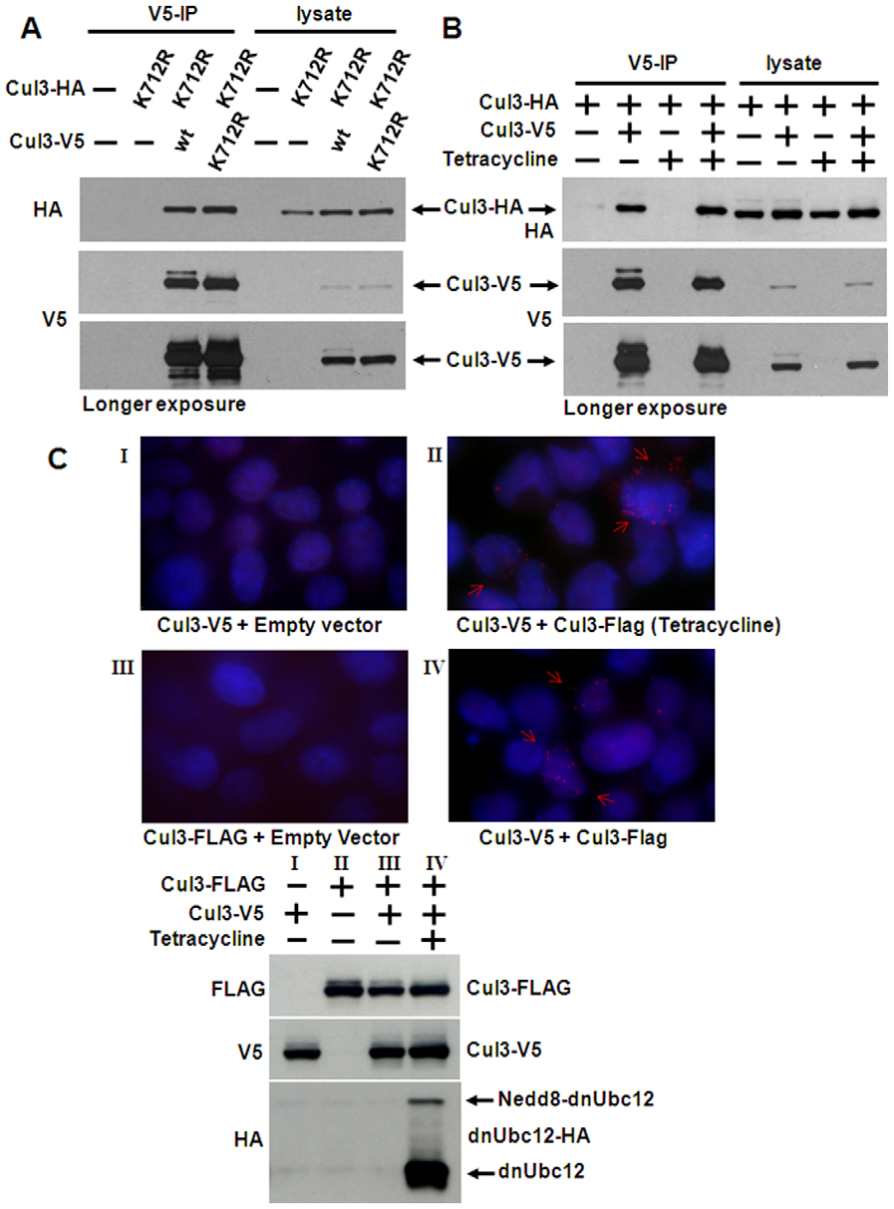
Inhibition of Cullin neddylation does not affect Cul3-Cul3 binding. *A*, The cells were cotransfected with Cul3(K712R)-HA and wild type Cul3-V5 or Cul3(K712R)-V5 as indicated at the top of the panel. The cells were lysed and the lysates subjected to V5 immunoprecipitation (*IP*). Immunoprecipitates and aliquots of the cell lysates were analyzed by Western blotting with the indicated antibodies. *B*, cells with stable expression of dnUbc12 under control of a tetracycline-inducible promoter were transfected in 60-mm cell culture plates for 2 days with expression constructs for the proteins indicated at the *top* of each panel, followed by 1 ìg/ml tetracycline treatment for 18 h, where indicated. The cell lysates were subjected to V5 immunoprecipitation (*IP*). Immunoprecipitates and aliquots of lysates were analyzed by Western blotting with the indicated antibodies. *C*, Tet-on dnUbc12 cells were transfected in 12-well cell culture plates as indicated at the *bottom* of each panel. Cells were treated with 1 µg/ml tetracycline for 24 h where indicated. The cells are fixed and were subjected to in situ proximity ligation assay, as described under “Materials and Methods” and then viewed by fluorescent microscopy. Aliquots of the cell lysates were analyzed by Western blotting with the indicated antibodies.

It has been reported that the Nedd8 molecule is essential for Cul3 dimerization and that Cul3 is a heterodimer consisting of one neddylated and one unneddylated Cul3 [28]. To confirm this, we used a Cul3 K712R mutant in which the neddylation site is mutated. Therefore this mutant cannot be conjugated with Nedd8. However, this mutation does not affect the interaction between Cul3 and BTB proteins [30]. We then co-transfected wild type or K712R mutant Cul3-V5 together with Cul3(K712R)-HA and performed V5 immunoprecipitation. As shown in Figure 3*A*, the binding affinity between two Cul3 K712R mutant proteins shows no difference compared to the binding affinity between K712R mutant and wild type Cul3. This result indicates that neddylation is not required for Cul3 dimerization.

To provide further evidence that cullin neddylation does not play a role in Cul3 dimerization, we used a HEK293 cell line with tetracycline-inducible expression of dominant negative, C111S mutant Ubc12 (dnUbc12) [27]. We co-transfected Cul3-V5 and Cul3-HA into dnUbc12 cells and induced the expression of dnUbc12 with tetracycline. Subsequently, we performed Cul3-V5 immunoprecipitation with the cell lysates to measure Cul3 self-association. As expected, induction with tetracycline resulted in loss of the neddylated form of Cul3 (Figure 3*B*). Consistent with our results in Figure 3*A*, there was no significant change in the Cul3-Cul3 binding between tetracycline induced and control cells (Figure 3*B*). In addition, we also visualized Cul3-Cul3 binding in the dnUbc12 cells by performing an *in situ* proximity ligation assay. In these experiments, Cul3-V5 and Cul3-Flag were cotransfected and the expression of dnUbc12 was induced with tetracycline. Two different primary and secondary antibodies directed against the V5 and Flag epitode tags were used and a ligation assay was performed. The signal from each ligated pair of Cul3-V5-Cul3-Flag dimers (or multimers) was detected by fluorescence microscopy. As shown in Figure 3*C*, the observed distinct fluorescent spots in control cells and in cells induced with tetracycline were qualitatively similar. Taken together, these results indicate that Nedd8 modification does not affect Cul3-Cul3 binding.

### The Cul3-Keap1 and Cul3-SPOP Complexes Contain Multiple Cul3 Proteins

**Figure 4:**
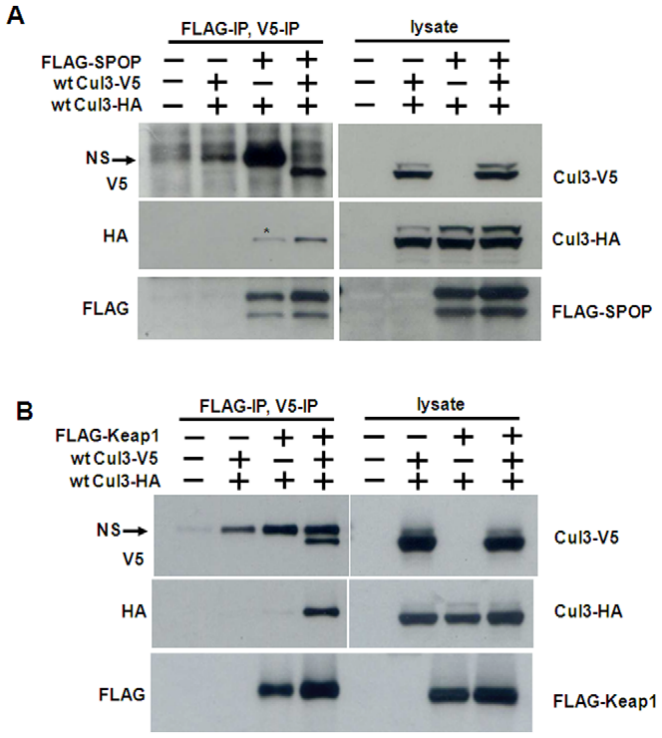
Multiple Cul3 proteins bind to Keap1 or SPOP dimers. *A–B*, The cells were transfected as indicated at the top of each panel. The cells were lysed, and the lysates were subjected to Flag immunoprecipitation (*IP*) and eluted with Flag peptide. The complexes were then subjected to V5 immunoprecipitation (*IP*). Immunoprecipitates and aliquots of the cell lysates were analyzed by Western blotting with the indicated antibodies. NS and * denote a non-specific band.

Small *et al*. (2010) showed that the recombinant Cul3/Rbx1-Keap1 complex is formed at a ratio of 1∶2 [31]. Thus, they reported that Cul3/Rbx1-Keap1 is a complex consisting of one molecule of each Cul3 and Rbx1 and two molecules of Keap1. In contrast, Zhuang *et al*. (2009) revealed that the recombinant SPOP protein forms a 2∶2 complex with Cul3/Rbx1 [17]. To determine the stoichoimetry of the Cul3 E3 ligase complexes *in vivo*, we co-transfected two differently tagged Cul3 proteins, Cul3-HA and Cul3-V5, together with Flag-SPOP or Flag-Keap1 into HEK293T cells. We then immunoprecipitated SPOP or Keap1 protein by using Flag agarose. The bound Cul3 complexes were eluted with Flag peptide. Subsequently, we performed V5-immunoprecipitation and probed the immunoprecipitates with HA antibody to detect tetrametric or multimeric Cul3-V5–Keap1–Keap1–Cul3-HA or Cul3-V5–SPOP–SPOP–Cul3-HA complexes. As shown in Figure 4*A*, both Cul3-V5 and Cul3-HA showed significant binding to Flag-SPOP. This result suggests that two or more Cul3 proteins are involved in the Cul3-SPOP complex *in vivo*. Similarly, both Cul3-V5 and Cul3-HA also exhibited significant binding to Flag-Keap1 (Figure 4*B*). These data therefore suggests that multiple Cul3/Rbx1 proteins are present in both the Cul3/Rbx1-Keap1 and the Cul3/Rbx1-SPOP complex *in vivo*. Thus, Cul3 E3 ligases exist at least partially in a dimeric or multimeric state containing substrate receptor dimers and multiple Cul3/Rbx1 proteins under *in vivo* conditions.

### Estimation of the proportion of Cul3 that exists in multimeric Cul3/Rbx1-BTB protein complexes *in vivo*

**Figure 5:**
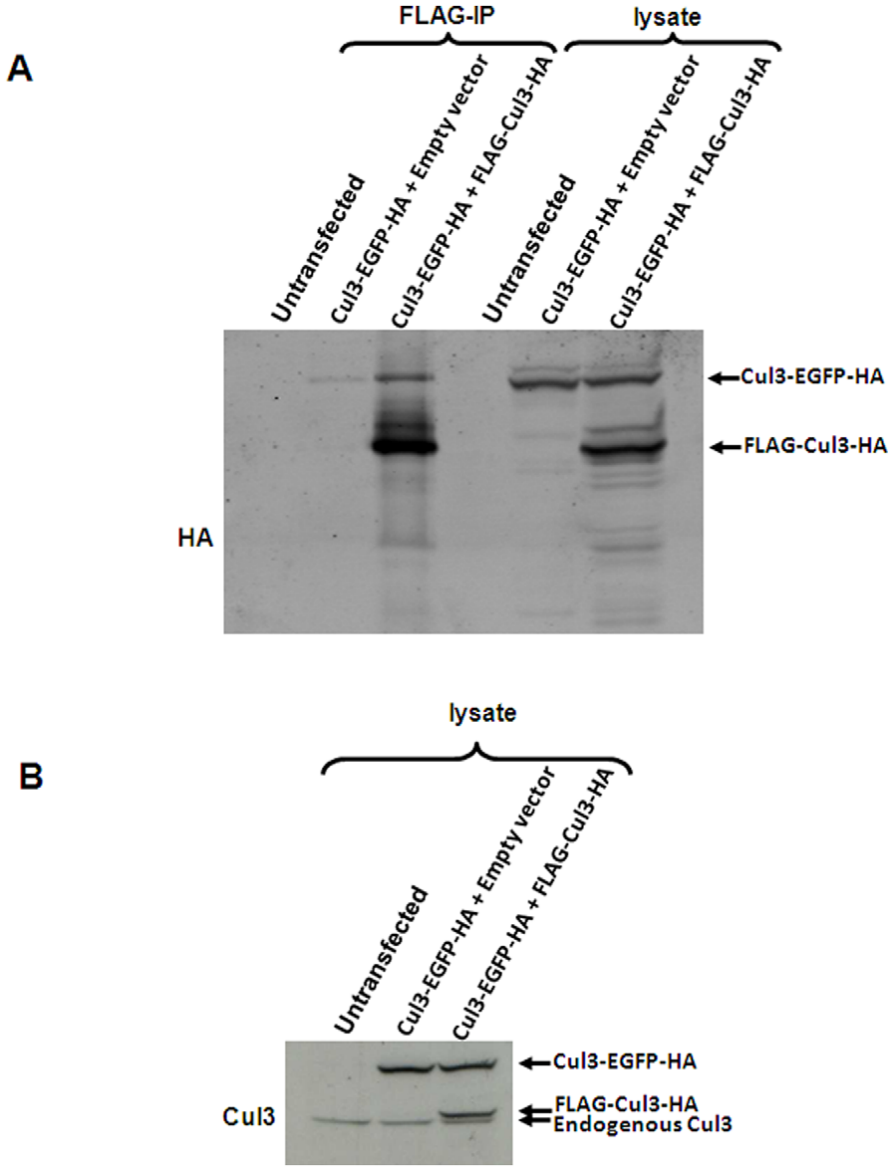
Estimation of the proportion of Cul3 that exists in a self-associated form *in vivo.* The cells were transfected as indicated at the top of each panel. The cells were lysed, and the lysates were subjected to Flag immunoprecipitation (*IP*). Immunoprecipitates and aliquots of the cell lysates were analyzed by Western blotting with the indicated antibodies. The quantification of the immunoprecipitates and aliquots of the cell lysates were analyzed by Western blotting with the indicated antibodies and digitized using the Odyssey Infrared Imaging system in the fluorescence intensity of 800CW, green.

The experiments described above indicate that Cul3 E3 ligases exist at least partially as tetrameric Cul3/Rbx1-BTB protein complexes. However, it is currently not clear what proportion of Cul3/Rbx1 exists as a dimer or multimer *in vivo*. To quantify the percentage of Cul3 that exists in a self-associated form, we expressed two forms of Cul3 with different molecular weights to distinguish them by their mobility in SDS-PAGE gels. One form consists of full length Cul3 with an N-terminal Flag tag that was used for immunoprecipitation. The other form consists of full length Cul3 carrying a C-terminal EGFP tag. To directly compare the abundances of the two Cul3 forms, both proteins also contained an HA tag at the extreme C-terminus. In our experimental approach, we co-transfected cells with both Cul3 forms followed by immunoprecipitation of Flag-Cul3-HA using Flag agarose. We then compared the ratio of Cul3-EGFP-HA to Flag-Cul3-HA in the cell lysates (input) with the ratio in the immunoprecipitates (corresponding to dimeric or multimeric Cul3 complexes). In the representative experiment shown in Figure 5*A*, the ratio of Cul3-EGPF-HA to Flag-Cul3-HA in the input (R_lysate_; lane 6) was 0.53∶1 whereas the ratio of Cul3-EGFP-HA to Flag-Cul3-HA in the immunoprecipitates (R_IP_; lane 3) was 0.18∶1. The percentage of Cul3-EGFP-HA bound to Flag-Cul3-HA can be calculated as R_IP_/R_lysate_×100% and amounts to 34%. In addition to Flag-Cul3-HA, Cul3-EGFP-HA can also associate with itself and with endogenous Cul3. The affinity of Cul3-EGFP-HA to itself, to Flag-Cul3-HA and to endogenous Cul3 is expected to be identical. Therefore, if the protein abundance of Cul3-EGFP-HA and Flag-Cul3-HA in the cell is equal, the amount of Cul3-EGFP-HA present in a dimer with Flag-Cul3-HA and with itself is also the same. As mentioned above, the ratio of Cul3-EGFP-HA to Flag-Cul3-HA in the input (lane 6) was 0.53∶1. It therefore follows that the percentage of Cul3-EGFP-HA that exists as a dimer with itself equals the percentage of Cul3-EGFP-HA/Flag-Cul3-HA dimer (i.e. 34%) multiplied by 0.53, resulting in 18%. To estimate the amount of Cul3-EGFP-HA bound to endogenous Cul3, we determined the ratio of endogenous Cul3 to Flag-Cul3-HA by Western blotting with Cul3 antibody. As shown in Figure 5*B*, the ratio is 0.41∶1. Therefore, the percentage of Cul3-EGFP-HA that exists as a dimer with endogenous Cul3 equals the percentage of Cul3-EGFP-HA/Flag-Cul3-HA dimer multiplied by 0.41, resulting in 14%. Adding the percentage of Cul3-EGFP-HA bound to itself (18%), to Flag-Cul3-HA (34%), and to endogenous Cul3 (14%) together results in a total of 66% of dimeric Cul3-EGFP-HA.

Three repeats of the experiment gave percentages of dimeric Cul3-EGFP-HA of 66%, 70% and 43% (average of 60.0%, standard deviation 14.6). It should be noted that the calculated percentage of Cul3-associated Cul3-EGFP-HA would not be affected by whether Cul3 forms dimeric or multimeric complexes *in vivo*. In the case of multimeric complexes, the percentage of Cul3-EGFP-HA bound to Flag-Cul3-HA would increase while the percentage of Cul3-EGFP-HA bound to only itself or only to endogenous Cul3 (and not to Flag-Cul3-HA) would concomitantly decrease. Taken together, the results suggest that approximately two thirds of cellular Cul3 protein exists in a self-associated form in Cul3/Rbx1-BTB protein complexes *in vivo*.

## Discussion

Dimerization is a key component of biological regulatory networks and is frequently employed in E3 ubiquitin ligases. For instance, RING finger protein 4 (RNF4), Anaphase Promoting Complex (APC) and C-terminal of Hsp70-interacting protein (CHIP) E3 ligases have been shown to exist in a dimeric state [32]–[34]. RNF4 is a RING E3 ligase which contains multiple SUMO-interaction motifs (SIMs). These SIMs specifically recognize poly-SUMO chains, thus targeting poly-SUMOylated substrate proteins for ubiquitination via the RNF4 RING domain [35]. The RNF4 dimerization is mediated via its RING domain. Dimerization promotes E2-ubiquitin thioester bond activation by recruiting the E2 to one RNF4, while the thioester-linked ubiquitin reaches across the dimer to engage the other RNF4 protein [32], [35]. APC, which is responsible for regulating the cell cycle has also been shown to exist as a dimer [33]. APC is a multisubunit E3 ligase and its dimerization is mediated via the Cdc27 subunit [33]. A Cdc27 mutant that is unable to homodimerize prevents the formation of the APC dimer, thereby leading to inhibition of the APC E3 ligase activity [33]. Thus, dimerization of APC is essential for its ligase activity.

Among the Cullin RING ligases (CRLs), Cul1, Cul3 and Cul4 based E3 ubiquitin ligases are known to exist as dimers [17], [26], [36]. Cullin serves as a scaffold protein for the assembly of the multisubunit ubiquitin ligase complex that contains a RING domain protein at its C-terminus and a cullin-specific substrate receptor protein at its N-terminus [10], [12]. Cul1 and Cul4 E3 ligase dimerization is mediated via the Cullin substrate receptors [17], [26], [36]. In the Cul1 E3 ligase, Cul1 recruits substrate recognition subunits containing a conserved F-box domain via the adaptor protein Skp1 to form Skp1-Cul1-F-box (SCF) E3 ligases. WD40 repeat F box proteins such as Cdc4 contain a D domain motif which mediates the homodimerization [26]. Each F box protein binds to one Cul1 protein via the Skp1 adaptor to form a dimeric SCF ligase complex [26]. It has been reported that a monomeric SCF^Cdc4^ complex is deficient in the ubiquitin transfer activity compared to a dimeric complex [26]. In the Cul4^DCAF1^ E3 ligase, dimerization is mediated via the DDB1-and Cul4-associated factor 1 (DCAF1) substrate receptor which contains a LisH motif [36]. LisH motif-mediated Cul4^DCAF1^ dimerization has been shown to increase the ubiquitin transfer activity by 2-fold compared to a monomeric complex [36]. There are 50 different DCAF substrate receptors for Cul4 in cells. However, the LisH motif is not commonly present among these putative DCAF substrate receptors. Therefore, it is currently not clear whether dimerization of DCAF proteins is a general mechanism to regulate Cul4 ligase activity.

In this study, we provide evidence that Cul3 E3 ubiquitin ligase dimerization is also mediated via substrate receptors. Cul3 substrate receptors contain a BTB domain which is known to homodimerize [13]. Homodimerization of BTB protein is important for substrate binding as described above. It has been proposed that homodimeric BTB proteins are also responsible for Cul3 dimerization by binding to two Cul3 proteins, forming a dimeric Cul3 E3 ligase complex [13]. In a previous study, we provided evidence that Cul3 indeed exists in a self-associated form which is dependent on the BTB protein substrate receptors [27]. However, Wimuttisuk and Singer, 2007 reported that two Cul3 proteins dimerize via their WH-B domains and that Cul3 dimers consists of one neddylated and one unnedylated Cul3. Thus, according to this model, the BTB proteins are not required for the Cul3 dimerization. In this study, we provide several lines of evidence that Cul3 dimerization is mediated via the BTB domain substrate receptor proteins and is independent of Cul3 neddylation. We found that Cul3 dimerization is dependent on the Cul3 N-terminus which interact with BTB substrate receptor proteins but not on the Cul3 C-terminus which contains the WH-B domain. Furthermore, Cul3 mutants that are unable to bind to BTB proteins exhibit markedly reduced Cul3 dimerization. Interestingly, some BTB proteins such as the Potassium channel tetramerization domain containing protein 11 (KCTD11) also form tetramers, raising the possibility of a tetrameric Cul3 E3 ligase complex. Indeed, modeling studies have shown that four Cul3 proteins could be accommodated in the structure of the KCTD11 tetramer [37]. Finally, we also showed that preventing Cul3 neddylation by mutating the neddylation site or using a neddylation-deficient cell line does not affect Cul3 dimerization. It should be noted that the low percentage of neddylated Cul3 and the high percentage of Cul3 that exists in a self-associated form, as determined in this study, would also be inconsistent with a model whereby Cul3 dimers consists of one neddylated and one unnedylated Cul3.

Cul3 E3 ligase dimerization likely plays an important role in promoting ubiquitination activity by providing a bivalent geometry that has two docking sites for the E2 ubiquitin enzyme and thus enhances ubiquitin transfer and elongation of the target substrate [17], [38]. Functional evidence for this has been provided by Kigoshi *et al*., (2011) who studied the function of the Cul3-KLHL7 E3 ligase [38]. The authors reported that a mutant of the KLHL7 BTB substrate receptor which is able to homodimerize with wild type KLHL7 but unable to bind to Cul3 inhibits ubiquitination of the target substrates. Thus, a heterodimeric Cul3 ligase complex consisting of wild type and mutant KLHL7 has reduced ubiquitination activity, suggesting that the presence of two bound Cul3 protein is necessary [38]. Zhuang *et al*. (2009) also showed that two Cul3 proteins are bound to two SPOP substrate receptors in the Cul3-SPOP structure [17]. They revealed that the recombinant Cul3 and SPOP form a 2∶2 complex. In contrast, Small *et al*. (2010) showed that a Keap1 homodimer only binds to a single Cul3 protein *in vitro* [31]. Our data as shown in Figure 4, indicate that two (or more) Cul3 proteins are bound to both the SPOP and Keap1 dimers *in vivo*. The difference between our results and those by Small *et al*. (2010) may be due to the difference in the expression system. Our results also suggest that the majority of the Cul3 proteins exist as a dimer or multimer in cells. Thus, BTB substrate receptor dependent dimerization or multimerization of Cul3 E3 ubiquitin ligases is likely of physiological significance in facilitating substrate polyubiquitination.

## Materials and Methods

### Plasmid constructs

The Flag-Cul3-HA and Cul3-EGFP-HA constructs were generated by insertion of the Cul3 ORF into the *Kpn*I and *Sac*II sites of modified pcDNA3.1/Zeo and pcDNA3, respectively. To generate C-terminally V5 tagged expression constructs for the N-terminal deletion construct of Cul3 (amino acids 392–768) and C-terminal deletion construct of Cul3 (amino acids 1–427), the respective cDNA was PCR amplified and inserted into the *Kpn*I and *Sac*II sites of modified pcDNA3. The (Cul2(NT)-Cul3(CT)-V5 was generated by fusing the N-terminus of Cul2 (amino acid 1–394)) to the C-terminus of Cul3 (amino acid 392–768) which also carrying a C-terminal V5-tag. Mutagenesis to prepare the K712R Cul3, and L52AE55A Cul3 mutants was carried out using the Stratagene site-directed mutagenesis kit. All other plasmids were previously described [27], [39]–[40].

The T-REx system (Invitrogen) was used to generate cell lines with tetracycline-inducible expression of dnUbc12-HA (dnUbc12) according to the manufacturer's instructions, as previously described [27]. For DNA transfections, sub-confluent T-REx-293 cells were transfected using GeneJuice (Novagen) according to the manufacturer's instructions.

### Immunoblotting

For immunoblotting, the cells were washed with ice-cold phosphate-buffered saline and then lysed in Triton X-100-containing lysis buffer, as previously described [41]. Lysates were precleared by centrifugation before use for Western blotting. Equal amounts of protein were loaded for Western blot analysis. The following antibodies were used: rabbit polyclonal anti-Cul3 (34–2200; Zymed Laboratories), monoclonal anti-V5 (AbD Serotec, Kidlington, UK), rat monoclonal anti-HA (clone 3F10) (Roche Applied Science).and monoclonal anti-FLAG M2 (Sigma-Aldrich, St. Louis, MO). Western blots shown are representative of at least two independent experiments. To quantify the dimerization of Cul3 in Fig. 5, the blot was imaged using an Odyssey Infrared Imaging system (LI-COR Biosciences, Lincoln NE). Sample amounts were determined and quantitated via IRDye 800CW in the 800 nm channel of detection.

### Immunoprecipitation

V5 antibody (2.5 µg), coupled to 20 µl of protein G-Sepharose (Amersham Biosciences, GE Healthcare, Waukesha, WI), or 20 µl of FLAG-agarose (Sigma) was used for immunoprecipitations, and 500 µl of precleared lysate from HEK293T cells transfected in 60-mm tissue culture plates was added. The samples were tumbled at 4°C for 2 h, and the beads were then washed four times in 1 ml of NP40 cold lysis buffer (containing 50 mM NaCl, 0.5% NP-40, 5% glycerol, 0.5 mM EDTA, 50 mM Tris, pH 7.5) and once in buffer containing 50 mM Tris (pH 7.5). The immunoprecipitated proteins were then denatured in SDS sample buffer and subjected to SDS–PAGE and Western blotting. The lysate lanes in the Western blots correspond to 5% of the input used for the immunoprecipitation. The immunoprecipitation experiments shown are representative of at least two independent experiments.

For the double immunoprecipitation in Fig. 4, FLAG immunoprecipitation was carried out as described above. After washing of the immunoprecipitates, the complexes were eluted with 30 µl Flag peptide (5 mg/ml) (F3290, Sigma), followed by V5 immunoprecipitation. The final immunoprecipitated proteins after washing were then denatured in SDS sample buffer and subjected to SDS–PAGE and Western blotting.

### In situ proximity ligation assay

In situ proximity ligation assay was performed using the Duolink detection 563 kit (Olink Biosciences, Uppsala, Sweden) following the manufacturer's instructions. Transfected cells were grown on coverslips. Cells were washed once with 1xPBS and fixed with 4% paraformaldehyde in PBS. TritonX-100 (0.1%) was used to permeabilize the fixed cells. As compatible antibodies for this particular experiment, Cul3-Flag was detected with a primary rabbit anti–Flag (Sigma) antibody while Cul3-V5 was detected using mouse anti–V5 (AbD Serotec) antibody. The corresponding probes of anti-rabbit PLUS and anti-mouse MINUS were from Olink Biosciences. Coverslips were mounted on glass slides using Vectashield mounting medium and visualized by fluorescence microscopy.
